# Surgery as a Potential Treatment Option for Patients With Stage III Small-Cell Lung Cancer: A Propensity Score Matching Analysis

**DOI:** 10.3389/fonc.2019.01339

**Published:** 2019-12-03

**Authors:** Chenyue Zhang, Cheng Li, Xiaoling Shang, Jiamao Lin, Haiyong Wang

**Affiliations:** ^1^Fudan University Shanghai Cancer Center, Fudan University, Shanghai, China; ^2^NHC Key Laboratory of Health Economics and Policy Research, School of Health Care Management, Shandong University, Jinan, China; ^3^Department of Dean's Office, Shandong Cancer Hospital and Institute, Shandong First Medical University and Shandong Academy of Medical Sciences, Jinan, China; ^4^Department of Clinical Laboratory, Qilu Medical College, Shandong University, Jinan, China; ^5^Department of Internal Medicine-Oncology, Shandong Cancer Hospital and Institute, Shandong First Medical University and Shandong Academy of Medical Sciences, Jinan, China

**Keywords:** surgery, small cell lung cancer, overall survival, SEER, prognosis

## Abstract

Surgery is commonly recommended for patients with stage I small-cell lung cancer (SCLC), whereas chemotherapy and radiotherapy are considered the standard treatment for patients with stage III SCLC. However, recent studies have suggested that a small proportion of patients with SCLC at an advanced stage may benefit from surgical resection. Therefore, in this study, we investigated the effectiveness of surgery in patients with stage III SCLC. Patients were selected from the Surveillance, Epidemiology, and End Results (SEER) database between 2004 and 2013. Propensity score matching (PSM) was used to eliminate any clinical bias. The overall survival (OS) was determined using the Kaplan–Meier method and compared using the log-rank test. The Cox proportional-hazards model was used to identify the effect of surgery on the OS. Of 9606 patients with stage III SCLC, 234 underwent surgery. Compared with the non-surgical group, a higher proportion of patients undergoing surgery had T1 and N0-N1 disease (risen by 10.7% for T1; 12.6% for N0-N1) and a lower proportion had T4 and N3 disease (decreased by 14.3% for T4; 12.5% for N3). The Kaplan-Meier analysis showed that patients who underwent surgery had a better OS before and after PSM. The multivariate analysis showed that surgery was beneficial for patients with stage III SCLC (HR: 0.651, 95% CI 0.524–0.808, *P* < 0.001). In conclusion, surgical resection might be associated with improved OS for patients with stage III SCLC and may be considered for the treatment of these patients. Further prospective studies are required to confirm these findings.

## Introduction

The incidence of lung cancer, which is the leading cause of cancer-related death worldwide, has been rising (1, 2). Small-cell lung cancer (SCLC), which accounts for about 15% of all lung cancer cases, is aggressive and has a tendency to metastasize (3–5). Consequently, SCLC has a dismal prognosis; <5% of the patients are diagnosed at stage I and most patients are diagnosed at advanced stages.

The treatment strategies of SCLC have changed over the past few years. Previously, surgical resection was recommended for limited stage SCLC. Recently, chemotherapy and radiotherapy are considered standard treatment for patients at advanced stages (6, 7). Indisputably, surgery is suitable for SCLC at the early stages. It has been reported that patients with stage III N2 disease can also benefit from surgery with subsequent chemotherapy or chemoradiotherapy (8). Besides, surgical resection of small lesions without lymph node involvement has been considered a curative strategy for patients with SCLC at an early stage. However, the benefit of surgery is limited to patients with SCLC at an early stage. An increasing number of studies have suggested that surgical resection can prolong the overall survival (OS) in patients with SCLC at an advanced stage (9, 10). Therefore, to further investigate the effect of surgery on patients with stage III SCLC, we performed a retrospective analysis using the Surveillance, Epidemiology, and End Results (SEER) database.

## Materials and Methods

### Data Collection

The SEER database is sponsored by the National Cancer Institute (NCI), which provides detailed information on the staging, baseline characteristics, and survival of patients with different types of cancer (11). We acquired the information using the official website of the database. We screened patients with stage III SCLC between 2004 and 2013 using the SEER^*^Stat software (version 8.3.5). We accessed the SEER study database via reference number 12356-Nov2017. The present study was approved by the ethics committee of Shandong Cancer Hospital and Institute. We adhered to the standard biosecurity and institutional safety procedures.

### Inclusion and Exclusion Criteria

The following data on patients diagnosed with stage III SCLC were collected from the SEER database: patients' age at diagnosis, gender, and race; the clinical characteristics included T stage, N stage, surgical treatment, and the use of radiotherapy. However, information on performance status (PS), comorbidities, type of hospital, and patient and surgeon attitude were inaccessible; hence, the relevant data were not recorded. Patients with survival and surgical records were included in this study. Patients with missing information regarding age, race, gender, T stage, N stage, surgery, and use of radiotherapy were excluded from this study. Furthermore, patients with additional cancer were also excluded.

### Propensity Score Matching (PSM)

PSM was conducted to eliminate any bias between the group of patients who underwent surgery (surgical group) and those who did not (non-surgical group). Using the chi-square test, the data on age at diagnosis, gender, race, T stage, N stage, and use of radiotherapy were included in the propensity model to generate a matching ratio of 1:1.

### Statistical Analysis

The SPSS software package, version 22.0 (IBM, SPSS Statistics, Chicago, IL, USA) was used to analyze the data. The chi-square test was used to analyze differences in clinical characteristics between the surgical and non-surgical groups. The Kaplan–Meier method was used to analyze the OS. The multivariate Cox proportional-hazards model was used to estimate the OS. A *P* value <0.05 was considered statistically significant.

## Results

### Clinicopathological Characteristics of Patients With Stage III SCLC

**Figure 1:**
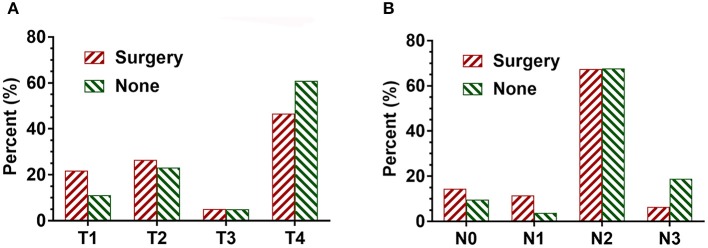
The proportion of T and N stage in SCLC patients with surgical and non-surgical treatments. **(A)** The distribution of T stage in SCLC patients with both surgical and non-surgical treatments. **(B)** The distribution of N stage in SCLC patients with both surgical and non-surgical treatments.

Overall 9,606 patients were diagnosed with SCLC. Of these, 234 (2.4%) patients underwent surgery, and the remaining 9,372 (97.6%) patients did not undergo surgery. There was no significant difference in age, gender, race, and use of radiotherapy between the patients in the surgical and non-surgical groups. However, there was a significant difference in T stage and N stage between the surgical and non-surgical groups. The baseline characteristics of the patients are shown in Table 1. Compared with the non-surgical group, there were more patients with T1 disease and fewer patients with T4 stage disease in the surgical group. Patients with T1 stage accounted for 21.8% in the surgery group whereas the percentage of T1 stage made up for 11.1 percent in the non-surgery group. Similarly, the proportion of patients with N0-N1 disease (26%) was higher in the surgical group than that in the non-surgical group (13.4%). In contrast, the proportion of patients with N3 disease (6.4%) was lower in the surgical group than that in the non-surgical group (Figure 1 and Table 1).

**Table 1:** Clinicopathological features of the SCLC patients at stage III.

**Characteristic**	**Surgery**	**No. surgery**	***P***
	**No. (%)**	**No. (%)**	
**Total**	234	9,372	
**Age**			0.497
< 65	106 (45.3)	4,037 (43.1)	
≥ 65	128 (54.7)	5,335 (56.9)	
**Sex**			0.606
Male	104 (44.4)	4,325 (46.1)	
Female	130 (55.6)	5,047 (53.9)	
**Race**			0.755
White	204 (87.2)	8,013 (85.5)	
Black	21 (9.0)	926 (9.9)	
Others	9 (3.8)	433 (4.6)	
**T stage**			< 0.001
T1	51 (21.8)	1,042 (11.1)	
T2	62 (26.5)	2,161 (23.1)	
T3	12 (5.1)	466 (5.0)	
T4	109 (46.6)	5,703 (60.9)	
**N stage**			< 0.001
N0	34 (14.5)	899 (9.6)	
N1	27 (11.5)	353 (3.8)	
N2	158 (67.5)	6,345 (67.7)	
N3	15 (6.4)	1,775 (18.9)	
**Radiation**			0.874
Yes	146 (62.4)	5,895 (62.9)	
No	88 (37.6)	3,477 (37.1)	

### Effect of Surgery on the OS in Patients With Stage III SCLC Before PSM

**Figure 2:**
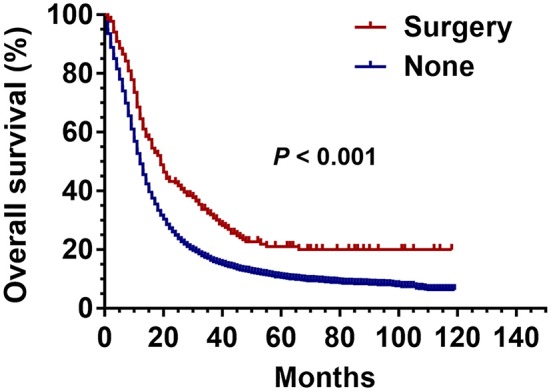
Kaplan Meier analysis for SCLC patients with surgical and non-surgical treatments.

The Kaplan-Meier analysis before PSM showed that the OS of patients with stage III SCLC was significantly better in the surgical group than that in the non-surgical group (*P* < 0.001) (Figure 2).

### Effect of Surgery on the OS of Patients With Stage III SCLC After PSM

**Figure 3:**
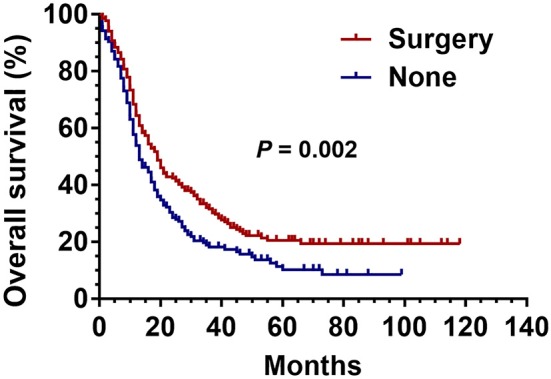
Kaplan Meier analysis for SCLC patients with surgical and non-surgical treatment after propensity matched analysis.

Before PSM, there were 234 patients in the surgical group and 9,372 patients in the non-surgical group. After 1:1 matching, each group included 234 patients; the ratio of patients with identical clinical characteristics was divided averagely between the two groups (Table 2). The Kaplan–Meier curve was generated after PSM, which revealed the advantage of surgery over non-surgical treatment in patients with stage III SCLC as shown in Figure 3 (*P* = 0.002).

**Table 2:** Propensity score matching (PSM) were conducted among SCLC patients with and without surgery at stage III.

**Characteristic**	**Surgery**	**No. surgery**
	**No. (%)**	**No. (%)**
**Total (1:1)**	234	234
**Age**		
< 65	106 (45.3)	106 (45.3)
≥ 65	128 (54.7)	128 (54.7)
**Sex**		
Male	104 (44.4)	104 (44.4)
Female	130 (55.6)	130 (55.6)
**Race**		
White	204 (87.2)	204 (87.2)
Black	21 (9.0)	21 (9.0)
Others	9 (3.8)	9 (3.8)
**T stage**		
T1	51 (21.8)	51 (21.8)
T2	62 (26.5)	62 (26.5)
T3	12 (5.1)	12 (5.1)
T4	109 (46.6)	109 (46.6)
**N stage**		
N0	34 (14.5)	34 (14.5)
N1	27 (11.5)	27 (11.5)
N2	158 (67.5)	158 (67.5)
N3	15 (6.4)	15 (6.4)
**Radiation**		
Yes	146 (62.4)	146 (62.4)
No	88 (37.6)	88 (37.6)

### Surgery as an Independent Prognostic Factor for Predicting the OS in Patients With Stage III SCLC

To evaluate the prognostic value of surgery in predicting the OS in patients with stage III SCLC, univariate analyses were performed; age, T stage, N stage, surgery, and use of radiotherapy were found to be important factors affecting the OS (Table 3). To avoid the interference, a multivariate analysis was conducted to determine the respective effect of these factors. The Cox proportional-hazards model confirmed that age, surgery, and use of radiotherapy were independent prognostic predictors of the OS in patients with stage III SCLC (Table 3).

**Table 3:** Surgery served as an independent prognostic factor in predicting OS among SCLC with stage III.

	**OS**		
**Variables**	**Univariate**	**Multivariate**	
	***P***	**HR (95CI%)**	***P***
**Age**	0.001		0.001
< 65		Reference	
≥ 65		1.433 (1.148–1.788)	0.001
**Race**	0.546		NI
White			
Black			
Other			
**Sex**	0.052		NI
Female			
Male			
**T stage**	0.044		0.047
T1		Reference	
T2		1.184 (0.865–1.622)	0.292
T3		1.503 (0.867–2.607)	0.147
T4		1.578 (1.138–2.190)	0.006
**N stage**	0.047		0.035
N0		Reference	
N1		1.410 (0.913–2.177)	0.121
N2		1.728 (1.198–2.491)	0.003
N3		1.520 (0.909-2.540)	0.110
**Surgery**	< 0.001		< 0.001
No		Reference	
Yes		0.651 (0.524–0.808)	
**Radiation**	< 0.001		< 0.001
Yes		Reference	
No		1.900 (1.513–2.386)	< 0.001

## Discussion

SCLC, one of the most lethal types of lung cancer, originates from pluripotent stem cells of the bronchial mucosal epithelium. However, the exact mechanism remains unclear. It is the worst differentiated type with malignancy. The doubling time of tumor cells of SCLC is as short as 23 days whereas that of squamous cell carcinoma and adenocarcinoma is 88 and 166 days, respectively. Therefore, it is a tricky disease in clinical practice. More than 60% of patients with SCLC are diagnosed at advanced stages, with a limited survival rate (12). It has been widely accepted that surgical resection is not appropriate for patients with SCLC at advances stages. Only a small proportion of patients with stage III SCLC undergo surgery; chemotherapy, and radiotherapy is considered more favorable treatment options for these patients (13). Therefore, the effectiveness of surgery in the treatment of patients with stage III SCLC has been controversial (14–16). In fact, a study conducted by Barnes underlined the drawbacks of surgery in limited-stage SCLC (17).

In the present study, therefore, we have incorporated a total of 9,060 patients diagnosed with stage III SCLC using the SEER database to address the above-mentioned confounding issue. We found that the patients who underwent surgery had a better survival outcome than those undergoing other treatments. The results of our study are consistent with that of some previous studies (9, 14). However, our findings are not consistent with conclusions from some studies on the effectiveness of surgery in SCLC treatment (18, 19).

A study conducted in 1973 compared the benefits of surgery and radiotherapy. The survival of patients with limited-stage SCLC who underwent surgery was found to be 6.5 months, which was far shorter than that of patients who underwent radiotherapy whose median survival reached 10 months (20). This study influenced the perception regarding the treatment strategy of SCLC among oncologists in the following 20 years, and has made surgery an inferior option.

The importance of surgery in the treatment early-stage SCLC has been widely acknowledged (19, 21, 22). And for SCLC patients at relatively advanced limited-stage, concurrent chemo-radiotherapy is greatly recommended (23, 24). Recent advances in radiology have enabled the early diagnosis of SCLC, and have prompted us to re-evaluate the effectiveness of surgery in SCLC.

Recently, many studies (25, 26) have demonstrated the encouraging results of SCLC patients with surgical treatment than those with other regimens. A study analyzed the information available on the National Cancer Database and showed a significant improvement in the 5-year survival rate (51%) of patients with stage I SCLC who underwent surgery compared with those who underwent other treatments (27).

Weksler evaluated the effect of surgical treatment in patients with SCLC using the SEER database; a total of 3,566 SCLC patients with stage I/II SCLC undergoing surgical treatment between 1988 and 2007 were included. Patients in the surgical group had a longer survival rate (25 months) than those in the non-surgical group (14 months) (25). The patients included in the study were diagnosed with limited-stage SCLC. Thus, it was concluded that surgical treatment was potentially beneficial for the treatment of early-stage SCLC.

Recently, several reports on the successful treatment of SCLC using surgical resection have been published (28, 29). However, due to lack of a control group and a relatively small sample size, the results have been considered less convincing.

Platinum-based chemotherapy with thoracic radiotherapy and prophylactic cranial irradiation (PCI) are used for the treatment of SCLC at early stages. Surgery was only recommended for patients with a solitary nodule and without hilar and mediastinal involvement and distant metastases (30). In a recent study conducted by Liu et al. it has been demonstrated that surgery-based multi-modality treatment is likely to improve the survival of patients with stage I SCLC and selected patients with II/III SCLC. Besides, comprehensive analyses have shown that the surgical technique may affect OS; patients undergoing lobectomy showed a better OS than with those undergoing sublobar resection (31).

In our study, the OS of patients in the surgical group significantly improved compared with those in the non-surgical group. It is possible that the patients in the non-surgical group initially achieve improvement because chemotherapy and radiotherapy kill the sensitive tumor cells; however, the insensitive tumor cells persist. This indicates artificial selection in the presence of chemotherapy and radiotherapy. These surviving tumor cells could be amplified thereafter, and subsequent treatment would be less effective. Therefore, the tumors could not be further treated with chemotherapy and radiotherapy. Most patients would relapse due to the proliferation of drug-resistant tumor cells. Meanwhile, the impairment of immunity (both humoral and cellular immunity) by the toxic drugs and radiation would prompt the proliferation of tumor cells (32, 33). Therefore, surgical treatment has been considered a favorable option in the treatment of SCLC. Thus, based on the results of our study, we recommend a broader application of surgical treatment in selected patients with stage III SCLC.

Several issues remain unanswered. It is not known whether surgery is necessary for patients with SCLC whose lesion is locally controlled after chemotherapy and radiotherapy. The appropriate time for patients with SCLC to undergo surgery remains unknown. Besides, the selection of chemotherapeutic drugs and surgical technique need to be addressed carefully.

There are several limitations in our study. First, it is a retrospective study, therefore, some characteristic of these patients were unavailable to obtain. Second, we only studied the effectiveness of surgery in the treatment of SCLC. However, we did not assess the combined effect of surgery with other treatments such as chemotherapy and radiotherapy. Third, some of the information such as PS, comorbidities, type of hospital, and patient and surgeon attitude were not available, and may influence the effect of surgery on the OS.

In conclusion, we showed that surgical treatment was linked to a better OS in patients with stage III SCLC. Therefore, we recommend the use of surgery in selected patients with stage III SCLC. Surgery, chemotherapy, and radiotherapy could be potentially well-coordinated to provide individualized treatment. However, further prospective studies are required to validate the effectiveness of surgery and its combination with other treatments in patients with SCLC.

## Data Availability Statement

The data used to support the findings of this study are available from the corresponding author upon request.

## Ethics Statement

The present study was approved by the ethics committee of Shandong Cancer Hospital affiliated to Shandong University.

## Author Contributions

CZ, CL, and HW conceived the study and wrote the paper. XS and JL performed analyzed the data. All authors approved the manuscript.

### Conflict of Interest

The authors declare that the research was conducted in the absence of any commercial or financial relationships that could be construed as a potential conflict of interest.
